# Special Issue: Cell Signaling and Molecular Pathology of Retinal Diseases: 2nd Edition

**DOI:** 10.3390/ijms27198730

**Published:** 2026-09-30

**Authors:** Hiroshi Tomita

**Affiliations:** Laboratory of Visual Neuroscience, Molecular Medical Science, Iwate University Division of Agriculture, 4-3-5 Ueda, Morioka 020-8551, Iwate, Japan; htomita@iwate-u.ac.jp; Tel.: +81-19-621-6427

Retinal degenerative diseases, a broad group encompassing age-related macular degeneration (AMD) and various forms of inherited retinal disease (IRD), are among the leading causes of irreversible blindness worldwide. Their global burden is expected to increase as populations age. AMD alone is estimated to affect nearly 200 million people worldwide, with this figure projected to reach approximately 288 million by 2040 [1]. Although individual IRDs are rare, collectively they affect approximately one in 2000 people and are a leading cause of blindness among working-age adults in several high-income countries [2,3]. Despite their diverse etiologies, these disorders converge on two principal cellular targets: photoreceptors and the retinal pigment epithelium (RPE). The dysfunction and eventual death of these cells ultimately lead to progressive loss of vision.

This Special Issue presents three papers presenting therapeutic approaches to tackle the key events of photoreceptor degeneration and two papers clarifying the molecular mechanisms of retinal degenerative diseases (Figure 1).

Puddu et al. [4] investigated whether saffron protects retinal pigment epithelial (ARPE-19) cells from hydrogen peroxide (H_2_O_2_)-induced oxidative damage, and whether its combination with Elderberry and Melilotus exerts synergistic beneficial effects. Oxidative stress is widely recognized as a critical driver of retinal photoreceptor degeneration. Mechanistically, Sanvicens et al. [5] demonstrated that the excessive accumulation of reactive oxygen species (ROS) triggers intracellular calcium influx and activates the calpain–caspase cascade, directly leading to photoreceptor apoptosis. Furthermore, Kanan et al. [6] revealed that tissue hyperoxia following rod cell loss subjects the remaining cone cells to severe oxidative stress, thereby disrupting their glucose metabolism and accelerating secondary cell death. These insights underscore the therapeutic importance of targeted antioxidant strategies to halt the progression of retinal degenerative diseases.

Consistent with these pathophysiological mechanisms, Puddu et al. [4] observed that H_2_O_2_ exposure significantly compromised cell viability and depleted glutathione levels, while concurrently elevating oxidative stress, ROS production, cell death markers, and inflammation. Notably, although pretreatment with saffron alone attenuated these damaging effects, the tri-herbal combination demonstrated superior efficacy in enhancing cell survival. Crucially, the authors elucidated the molecular mechanisms underlying this cytoprotective effect: the combination treatment activated the NRF2 pathway and its downstream antioxidant enzyme, superoxide dismutase 2 (SOD2), while suppressing both the activation of the inflammatory enzyme caspase-1 and the subsequent production of the cytokine interleukin-1β (IL-1β). These findings align with the broader historical context of nutritional interventions for retinal diseases—most notably the landmark AREDS2 trial, which evaluated the capacity of antioxidant supplementation and omega-3 fatty acids to slow the progression of age-related macular degeneration (AMD) [7,8].

In contrast to Puddu et al., [4] who targeted upstream ROS generation, Tahia et al. [9] focused on downstream sphingolipid metabolism in retinal degeneration. Since ceramide is a well-established mediator of photoreceptor apoptosis [10,11], they showed that L-cycloserine, a blocker of the first step in ceramide production, effectively rescues photoreceptors and preserves both retinal structure and function. This comprehensive study tracks drug distribution to the retina, evaluates tissue lipid profiles, and monitors downstream gene and protein expression. What makes this approach conceptually significant is its gene-agnostic logic: because ceramide-driven cell death is a final common pathway in multiple retinal degenerative diseases [12], targeting it could benefit patients independent of their underlying genetic causes, contrasting sharply with gene-by-gene therapies. However, the protective effect operated within a narrow window; a moderate dose was beneficial, while a higher dose was not. This highlights the need for finely tuned modulation of ceramide signaling to restore cellular homeostasis rather than completely suppressing it. This in vivo neuroprotection was further substantiated by the fact that intraperitoneally administered L-cycloserine rapidly reaches the retina, brain, liver, and plasma within an hour. Its efficacy was robustly validated through ERG and histological analyses. Mechanistically, L-cycloserine attenuates light-induced damage by suppressing ceramide biosynthesis and mitigating the upregulation of Cathepsin D, a prominent lysosomal protease in non-caspase apoptotic pathways. By collectively intercepting ceramide accumulation and the subsequent Cathepsin D-mediated cascade, L-cycloserine highlights its promising therapeutic potential against light-induced retinal injury.

Furthermore, a compelling narrative emerges when linking the ceramide-mediated apoptotic pathway with the pathophysiological role of fatty acid-binding proteins (FABPs). Ohguro et al. in their review [13] reported that intraocular levels of FABP4 (ioFABP4) and free fatty acids (ioFFA) are significantly elevated in retinal vascular diseases (RVDs) [14], such as proliferative diabetic retinopathy (PDR) and retinal vein occlusion (RVO). Crucially, their work argues that ioFABP4 and its substrates (ioFFAs) are produced locally within the eye—with the choroid being a likely source—rather than entering from the systemic circulation. Given that FABPs function as lipid chaperones that facilitate the intracellular trafficking of long-chain saturated fatty acids like palmitate [15], they effectively provide the precise, rate-limiting substrates required for the initial step of de novo ceramide biosynthesis. Because L-cycloserine provides robust neuroprotection by blocking this initial biosynthetic step, it is plausible that elevated ioFABP4 in RVDs accelerates retinal degeneration by overloading photoreceptors with locally generated FFAs, fueling the downstream, pro-apoptotic ceramide cascade and subsequent Cathepsin D upregulation [16]. Importantly, this local lipid-handling system extends beyond a single protein or pathway. Ohguro et al. [13] also noted that a related isoform, FABP5, is elevated in RVDs, though it exhibits a distinct pathological pattern with a more pronounced increase in RVO than in PDR. Most significantly, FABP4 expression appears to be regulated independently of vascular endothelial growth factor (VEGF) [17]. Because current anti-VEGF therapies fail to benefit every patient and impose a heavy clinical burden due to the necessity of repeated injections, an independently regulated target such as FABP4 presents a highly appealing therapeutic prospect. By implicating a localized lipid-handling network rather than an isolated angiogenic factor, the review of Ohguro et al. [13] bridges the lipid-metabolic theme of retinal degeneration directly into the vascular arena. Consequently, simultaneously targeting the upstream lipid chaperone FABP4 and downstream sphingolipid metabolism could offer a synergistic, multi-tiered therapeutic paradigm for treating diverse, complex retinal degenerative and vascular diseases.

In their review, Jiang et al. [18] provide a comprehensive conceptual framework for the cellular and molecular signaling mechanisms driving dry and wet age-related macular degeneration (AMD) subtypes. Rather than treating pathological events as isolated findings, their synthesis details how oxidative stress, NLRP3 inflammasome-mediated inflammation (including interleukin-1β release), complement dysregulation, disturbed lipid handling, and VEGF-driven angiogenesis interact within a connected pathological system. Crucially, by mapping these common molecular pathways and their cross-talk, including the NRF2 antioxidant system and early-stage lipid deposits, Jiang et al. [18] offer a vital framework for interpreting focused experimental studies as integrated components of a broader disease network. A prime example of this integrated network is the complement dysregulation driven by the CFH Y402H polymorphism (rs1061170) [19,20], which is prevalent in approximately 50% of AMD patients. Jiang et al. [18] highlight how this specific genetic variant serves as a multifactorial driver of AMD pathogenesis by profoundly disrupting metabolic homeostasis, lipid metabolism, and oxidative stress responses. Mechanistically, the Y402H variant impairs the regulatory and protective capacities of CFH, leading to uncontrolled C3b deposition and membrane attack complex (MAC) formation, which ultimately results in RPE lysis and photoreceptor degeneration [21]. Crucially, this complement amplification—further exacerbated by key gain-of-function variants in CFB and C3—synergizes with altered lipid handling, accelerating lipoprotein accumulation in drusen and amplifying localized tissue damage. Ultimately, this cascade bridges genetic susceptibility, chronic inflammation, and tissue remodeling, positioning complement dysregulation as both a pivotal biomarker and a promising therapeutic target in AMD.

Alibrandi et al. [22] address one of the most challenging problems in retinal genetics: families whose disease does not conform to an expected single-gene pattern. They focus on two rare variants found together in affected individuals: one in AIPL1, a gene essential for photoreceptor protein folding, and another in BBS2, which is involved in intracellular transport. Using a layered computational approach incorporating protein structure prediction, molecular modeling of protein interactions, and analysis of broader gene networks, the authors propose that these variants may act as a digenic pair, jointly causing disease that neither variant would cause alone. The authors appropriately present this as a hypothesis rather than a confirmed mechanism, as their findings are based entirely on computational analyses and require experimental validation. Nevertheless, this cautious interpretation is one of the study’s strengths. More broadly, the study illustrates how the field can begin to account for patients who remain genetically unexplained and highlights a practical lesson: when single-gene testing fails to identify a cause, it may be necessary to consider how multiple variants interact. From genetic origins, the focus shifts to disease mechanisms.

In conclusion, the strength of this Special Issue lies not in any single finding but in the broader picture that emerges when its contributions are considered together. They suggest that the progression from a damaged gene to retinal blindness involves a surprisingly small number of shared molecular pathways. It is along these shared pathways that diagnosis can become more precise and treatment more broadly applicable, while advances in gene therapy, disease modeling, and artificial intelligence offer further opportunities for meaningful progress in retinal research and patient care.

**Figure 1:**
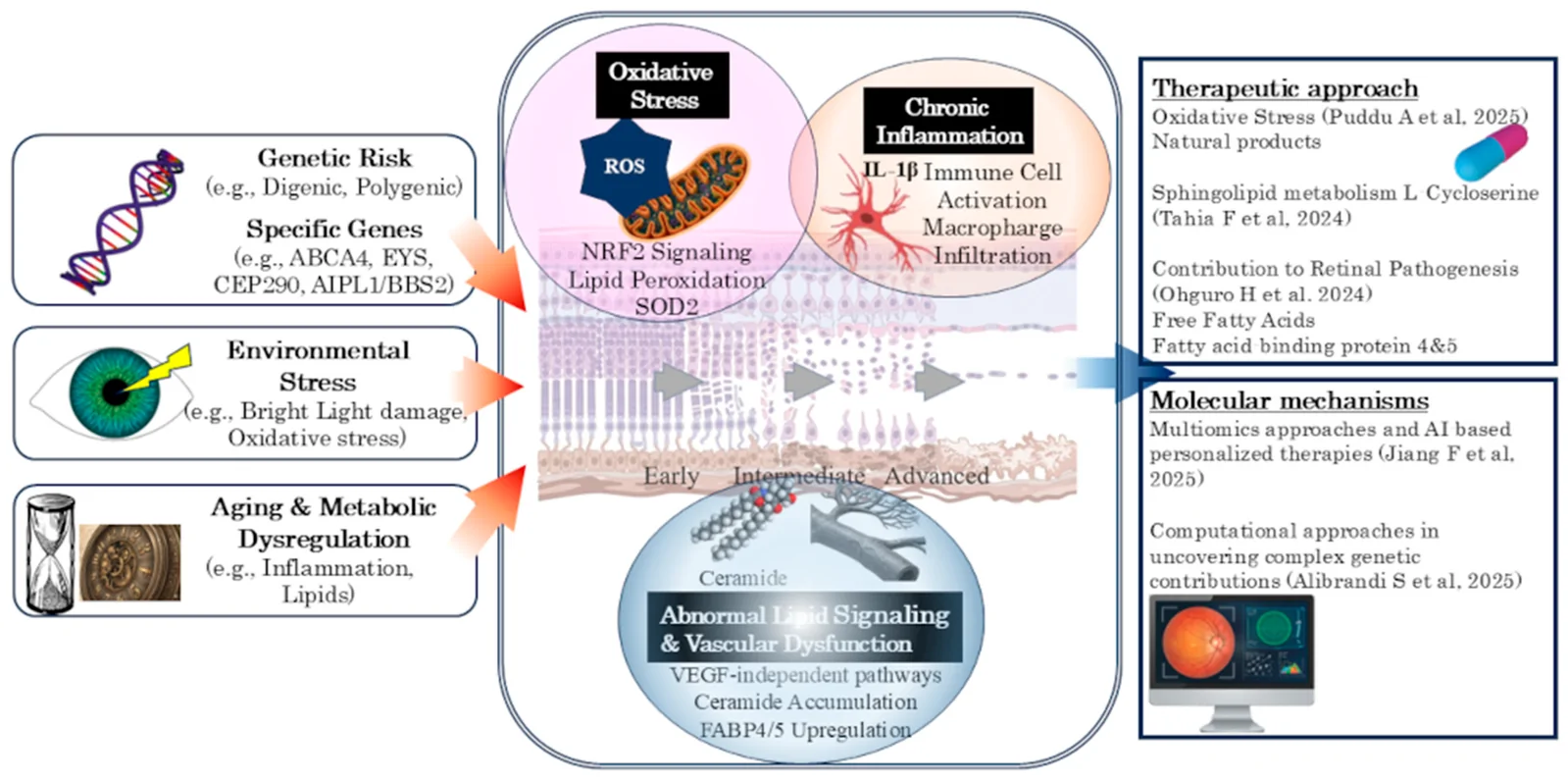
Schematic overview of the Special Issue: Cellular and molecular pathways in retinal degeneration and advancing therapeutic strategies. The diagram illustrates the interconnected pathogenic mechanisms associated with inherited retinal dystrophies, retinitis pigmentosa, and age-related macular degeneration (AMD), alongside targeted therapeutic interventions. Pathogenesis: Retinal degeneration results from the convergence of genetic and environmental stressors. Digenic variants (e.g., AIPL1 and BBS2) disrupt photoreceptor proteostasis, while elevated intraocular FABP4 (ioFABP4) and free fatty acids promote pathogenic lipid signaling. Concurrent accumulation of ceramide contributes to photoreceptor apoptosis. In the retinal pigment epithelium, oxidative stress and mitochondrial dysfunction induce severe inflammatory responses (characterized by elevating Caspase-1/3 and interleukin-1β), contributing to dry AMD (including drusen formation and geographic atrophy) or vascular endothelial growth factor (VEGF)-mediated angiogenesis in wet AMD. Therapeutic approaches: Emerging interventions target these pathogenic cascades. L-cycloserine protects photoreceptors by inhibiting de novo ceramide synthesis, while a saffron–elderberry–melilotus mix mitigates oxidative damage via activation of the nuclear factor erythroid 2–related factor 2 (NRF2) pathway. In parallel, multi-omics approaches and artificial intelligence (AI) accelerate target discovery (including the identification of ioFABP4), thereby supporting the development of personalized therapeutic strategies [4,9,13,18,22].

## Abbreviations

The following abbreviations are used in this manuscript:

AI	Artificial intelligence
AMD	Age-related macular degeneration
FABP4	Fatty acid-binding protein 4
FABP5	Fatty acid-binding protein 5
ioFABP4	Intraocular fatty acid-binding protein 4
IRD	Inherited retinal disease
NRF2	Nuclear factor erythroid 2–related factor 2
RPE	Retinal pigment epithelium
SOD2	Superoxide dismutase 2
VEGF	Vascular endothelial growth factor
